# Comparison of Different Magnetic Resonance Cholangiography Techniques in Living Liver Donors Including Gd-EOB-DTPA Enhanced T1-Weighted Sequences

**DOI:** 10.1371/journal.pone.0113882

**Published:** 2014-11-26

**Authors:** Sonja Kinner, Verena Steinweg, Stefan Maderwald, Arnold Radtke, Georgios Sotiropoulos, Michael Forsting, Tobias Schroeder

**Affiliations:** 1 Department of Diagnostic and Interventional Radiology and Neuroradiology, University Hospital Essen, Essen, Germany; 2 Erwin L. Hahn Institute for Magnetic Resonance Imaging, University Duisburg Essen, Essen Germany; 3 Department of General, Visceral and Transplantation Surgery, University Hospital Essen, Essen, Germany; The First Affiliated Hospital of Nanjing Medical University, China

## Abstract

**Objectives:**

Preoperative evaluation of potential living liver donors (PLLDs) includes the assessment of the biliary anatomy to avoid postoperative complications. Aim of this study was to compare T2-weighted (T2w) and Gd-EOB-DTPA enhanced T1-weighted (T1w) magnetic resonance cholangiography (MRC) techniques in the evaluation of PLLDs.

**Materials and Methods:**

30 PLLDs underwent MRC on a 1.5 T Magnetom Avanto (Siemens, Erlangen, Germany) using (A) 2D T2w HASTE (Half Fourier Acquisition Single Shot Turbo Spin Echo) fat saturated (fs) in axial plane, (B) 2D T2w HASTE fs thick slices in coronal plane, (C) free breathing 3D T2w TSE (turbo spin echo) RESTORE (high-resolution navigator corrected) plus (D) maximum intensity projections (MIPs), (E) T2w SPACE (sampling perfection with application optimized contrasts using different flip angle evolutions) plus (F) MIPs and (G) T2w TSE BLADE as well as Gd-EOB-DTPA T1w images without (G) and with (H) inversion recovery. Contrast enhanced CT cholangiography served as reference imaging modality. Two independent reviewers evaluated the biliary tract anatomy on a 5-point scale subjectively and objectively. Data sets were compared using a Mann-Whitney-U-test. Kappa values were also calculated.

**Results:**

Source images and maximum intensity projections of 3D T2w TSE sequences (RESTORE and SPACE) proved to be best for subjective and objective evaluation directly followed by 2D HASTE sequences. Interobserver variabilities were good to excellent (k = 0.622–0.804).

**Conclusions:**

3D T2w sequences are essential for preoperative biliary tract evaluation in potential living liver donors. Furthermore, our results underline the value of different MRCP sequence types for the evaluation of the biliary anatomy in PLLDs including Gd-EOB-DTPA enhanced T1w MRC.

## Introduction

Adult-to-adult Living donor liver transplantation (ALDLT) is a widely established surgery option for end-stage liver disease [1], [2]. Particularly the right graft ALDLT is still burdened with a high up to 32% incidence of biliary complications in the recipients [3], [4] Anastomotic complications in the graft can be minimized if detailed knowledge of the biliary anatomy of donors is present. Anatomic variations especially of the right-sided intrahilar bile duct system are frequent and the knowledge of variations can decisively influence the surgical approach [5]–[7]. A preoperative 3D-Cholangio-MRI has been widely used to evaluate the biliary anatomy of the potential living liver donor (PLLD) in ALDLT nearly completely replacing the endoscopic retrograde cholangiopancreatography (ERCP) as the reference standard at the most centres because of its considerable peri- or post-interventional risks including cholangitis, bleeding and biliary perforation [8], [9].

Contrast enhanced computed tomography cholangiography (ce-CTC) using meglumine iotroxate (Biliscopin, Bayer Schering Pharma, Berlin, Germany) has become a non-invasive alternative imaging method prior to surgery and was a reference standard for the preoperative donor selection in our institution [10]–[12]. At most centres the intraoperative cholangiography has still been applied prior to the graft hepatectomy in order to 1) verify the intrahilar biliary anatomy and to 2) navigate the surgeon placing the clip inside the hilar plate (HP) as an landmark for the definitive trans-hilar passage [13]. As PLLDs are mostly young and otherwise healthy patients, an imaging method without the need of iodizing radiation would be the optimal screening tool. Furthermore, meglumine iotroxate has been taken from the market. Over the years, magnetic resonance cholangiography (MRC) has evolved as a non-invasive surrogate for ERCP and ce-CTC [14]. Considerable refinements of MRC techniques have been achieved during the last two decades including higher spatial resolution. Furthermore, with the introduction of hepatocyte specific contrast agents, which show a considerable secretion over the bile, contrast-enhanced T1-weighted techniques have been proposed and evaluated [15], [16]. Some of these techniques have also been tested and compared in the evaluation of PLLDs [17], [18]. However, until now, there is no general agreement on which MRC technique provides the highest diagnostic accuracy for the visualisation of biliary anatomy.

The aim of this study therefore was to compare different MRC sequences including Gd-EOB-DTPA enhanced T1-weighted sequences with regard to the display of the biliary tract and to compare it to ce-CTC as reference standard.

## Materials and Methods

### Patients and Imaging

The study was approved by the local institutional review board, ethics committee university hospital Essen. Written informed consent was obtained from all patients. A total of 30 potential living liver donors (18 men, 12 women, age: 20–60 years, mean age 34 years) underwent meglumine iotroxate-based computed tomography cholangiography (contrast enhanced CTC; ce-CTC) as a part of preoperative workup for right graft ALDLT and were willing to take part in the study. Twelve candidates eventually underwent surgery for right graft donation.

The ce-CTC data sets served as reference standard pre- and intraoperatively while for operated patients ce-CTC as well as intraoperative choloangiography served as reference standards.

### Contrast enhanced CTC protocol and 3-dimensional CT-image analysis

CT imaging and 3-D reconstructions were performed using a 16-row-Multidetector-CT-Scanner (Sensation16, Siemens, Germany) and the software assistant HepaVision (MeVis, Bremen, Germany) as already published [12].

### MR Cholangiography

One to four days after the initial CT scan all 30 patients underwent MR imaging on a 1.5 T MR (Magnetom Avanto, Siemens, Erlangen Germany). Neither spasmolytic agents nor oral contrast agents were administered. A torso phased-array surface coil and the integrated spine array coil (total number of 12 coil elements) were used for signal reception. The following MRCP sequences were acquired: (A) 2D T2w HASTE (Half Fourier Acquisition Single Shot Turbo Spin Echo) fat saturated (fs) in axial plane, (B) 2D T2w HASTE fs thick slices in coronal plane, (C) free breathing 3D T2w TSE (turbo spin echo) RESTORE (high-resolution navigator corrected) plus (D) maximum intensity projections (MIPs), (E) T2w SPACE (sampling perfection with application optimized contrasts using different flip angle evolutions) plus (F) MIPs and (G) T2w TSE BLADE (vendor (Siemens) specific name for PROPELLER = periodically rotated overlapping parallel lines with enhanced reconstruction sequence). While sequence A, B and G were acquired using breath-hold techniques, sequences C–F were collected using navigator correction. This technique can reduce motion artefacts by directly monitoring the right diaphragm with navigator echoes. Parallel imaging (PAT factor 2) was applied for all sequences. 20 minutes after contrast injection (Gd-EOB-DTPA, Bayer, Berlin, Germany) 3D T1-weighted FLASH (fast low angle shot) sequences (H) without and (I) with inversion recovery (IR) pulse were acquired in axial planes. Coronal reconstructions of these data sets were performed. Imaging parameters of all nine compared MRCP sequences are listed in Table 1.

**Table 1:** Imaging parameters of the nine compared MRCP sequences.

		TR[ms]	TE[ms]	Flip angle[deg]	Matrix	Slicethickness[mm]	Acquisitiontime [s]	Echo trainlength
A	2D HASTE ax	700	117	150	192	5	32	256
B	2D HASTE cor	4500	754	180	349	40	2	256
C (D)	3D TSE RST (MIP)	3414	638	180	380	2 (80)	240–400(*)	127
E (F)	3D SPACE (MIP)	3670	650	140	380	1 (72)	180–400(*)	135
G	2D TSE BLADE	8660	93	150	384	6	220–360(*)	29
H	3D ce-T1w FLASH ax	3.35	1.15	15	384	2	22	1
I	3D ce-T1w FLASH ax +IR	400	1.5	10	320	2	36	1

(*) depending on patients’ breathing.

### The virtual trans-hilar passage – anatomical definitions

We defined the **right sided hilar corridor** as the narrow intrahepatic space for the hilar transection line within the hilar plate (HP), between the central hilar = 1° biliary branching level (**medial boundary**) and the right peripheral = 2°- sectorial/3°- segmental branching levels (**lateral boundary**), as already proposed [12].

According to the Couinaud classification as modified by Smadja et Blumgart bifurcations were regarded as “normal” (N) hilar anatomy while trifurcations and quadrifurcations were considered “abnormal” (A) biliary anatomy [19].

### Data evaluation

Two consultant radiologists assessed the images independently. The radiologists were unaware of the results of CTC. MRC sequences were evaluated separately in a randomized order. The biliary display was rated quantitatively on the basis of a 5-point Likert scale, ranging from 1 = ‘bile ducts apparent up to 3° branching level of the left and right segmental hepatic duct’ to 5 = ‘no bile duct system visible’ (see Table 2). Qualitative analysis scored the depiction of the biliary tract visualized on ce-CTC and ce-MRC data sets (T1w with and without IR) as related to the final intraoperative findings in n = 12 operated donors and was also based on a 5-point scale, ranging from 1 = ‘excellent image quality/clearly distinguishable bile ducts’ to 5 = ‘non- diagnostic image quality (see Table 2).

**Table 2:** Scores used for quantitative and qualitative analysis of cholangiographies.

	Quantitative analysis	Qualitative analysis
**Score 1**	left and right segmental hepatic ducts apparent(3° branching level)	*excellent image qualityand highest confidence level*
**Score 2**	left and right sectorial hepatic duct apparent(2° branching level)	high image quality with high diagnostic validity and high confidence level
**Score 3**	left and right main hepatic duct apparent(1° branching level)	mean image quality and mean diagnostic validity
**Score 4**	Solely common hepatic duct visible	worse image quality with very limited diagnostic validity and only limited confidence
**Score 5**	*no bile duct system visible*	*non-diagnostic image quality*

### Statistical Analysis

IBM SPSS Statistics for Windows, Version 20.0 (IBM Corp., Armonk, NY, USA) was used for statistical analysis. A Wilcoxon test was used to correlate different MRC sequences. A p value <0.05 indicated statistically a significant difference.

To estimate interobserver agreement for subjective and objective evaluation of the biliary tract for all MRC sequences, a Kappa analysis was performed (k <0.20 indicated poor agreement; k of 0.21–0.40 fair agreement; k of 0.41–0.60 moderate agreement; k values of 0.61–0.80 good and k>0.8 excellent interobserver agreement).

## Results

All patients underwent ce-CTC and MRC including contrast enhanced T1-weighted MRC without any noticeable adverse events. Ce-CTC and all MRC sequences were of diagnostic image quality and the biliary tract was visible and evaluable.

### Quantitative evaluation

**Figure 1:**
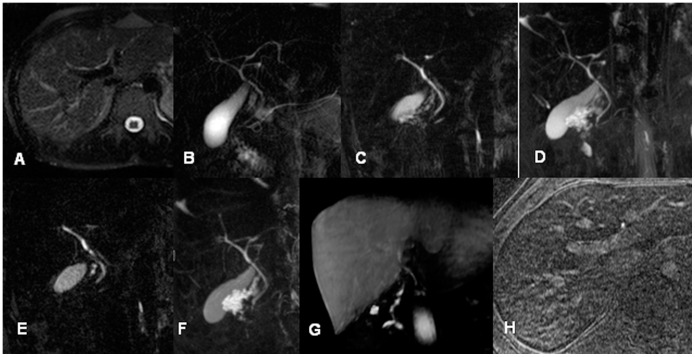
Comparison of MRC sequences in a 26-year-old male potential living liver donor (A: axial T2w HASTE; B coronal thick slab HASTE; C: central single image of coronal T2w 3D RESTORE; D: MIP of C; E: central single image of coronal T2w 3D SPACE; F: MIP of E; G: Gd-EOB-DTPA enhanced T1w FLASH sequence (coronal MPR) and H: axial Gd-EOB-DTPA enhanced T1w FLASH with IR pulse). Note that all MRC data show abnormal central anatomy with Smadja & Blumgart Type D1 trifurcation while 3D sequences and coronal HASTE provide insight in the more peripheral bile ducts with drainage of the right posterior segment 7 duct into the left main hepatic duct.

Bile ducts were best visualized using the T2 TSE 3D RESTORE sequence (mean value 2.35+/−0.11) and the calculated MIP (mv: 2.5+/−0.11). The following preferred MRCP sequences were T2 SPACE MIP (mv: 2.51+/−0.15) and source images (mv: 2.57+/−0.15). For T2 HASTE images the mean value was 2.57+/−0.09. Contrast-enhanced T1w MRC images were rated with mean values of 2.8+/−0.11 and 2.92+/−0.12 using inversion recovery. Axial HASTE sequences were rated with a mean value of 2.95+/−0.09. Worst results were found for the T2 TSE BLADE sequence fat saturated (mv: 3.75+/−0.2) which provided only limited information on the biliary anatomy. A comparison of the separate sequences concerning statistical significance can be found in Table 3. Image impression of the sequences is shown in Figure 1.

**Table 3:** Interindividual comparison of MRCP sequences concerning I) quantitative evaluation and II) qualitative evaluation: S: statistical significant difference.

I)	A	B	C	D	E	F	G	H	I
A		0,001 S	<0,001 S	0,002 S	0,034 S	0,014 S	0,016 S	0,255	0,926
B			0,015 S	0,315	0,759	0,292	0,005 S	0,044 S	0,014 S
C				0,001 S	0,070	0,404	0,002 S	0,004 S	<0,001S
D					0,750	0,543	0,003 S	0,066	0,004 S
E						0,084	0,005 S	0,162	0,007 S
F							0,002 S	0,080	0,004 S
G								0,015 S	0,015 S
H									0,519
**II)**	**A**	**B**	**C**	**D**	**E**	**F**	**G**	**H**	**I**
A		0,001 S	<0,001 S	<0,001 S	0,004 S	0,002 S	0,042 S	0,830	0,591
B			0,049 S	0,246	0,412	0,248	0,005 S	0,012 S	0,001 S
C				0,005 S	0,490	0,623	0,003 S	0,002 S	<0,001 S
D					0,873	0,729	0,003 S	0,007 S	<0,001 S
E						0,705	0,003 S	0,031 S	0,001 S
F							0,001 S	0,025 S	0,001 S
G								0,016 S	0,038 S
H									0,594

A: 2D HASTE axial; B: 2D HASTE coronal; C: 3D TSE RESTORE; D: MIP of C; E: 3D TSE SPACE; F: MIP of E; G: 2D BLADE axial; H: Gd-EOB-DTPA enhanced T1w FLASH; I: Gd-EOB-DTPA enhanced T1w FLASH with IR.

### Qualitative evaluation

Of all nine magnetic resonance cholangiography (MRC) images source images of 2D TSE RESTORE sequence and T2 SPACE MIP revealed best results concerning qualitative evaluation (mv: 2.3+/−0.12). Source images of T2 SPACE showed a mean value of 2.35+/−0.16; MIP images of the T2 RESTORE sequence a mean value of 2.43+/−0.1. The thick slab HASTE sequence reached a mean value of 2.56+/0.1. Contrast-enhances T1w MRC sequences with and without IR reaches mean values of 2.88+/−0.12 and 2.9+/0.11. The axial HASTE sequence was rated with a mean value of 2.95+/−0.11. Worst results were found for the axial BLADE sequence with a mean value of 3.67+/−0.22. A comparison of the separate sequences concerning statistical significance can be found in Table 3.

### Detection of anatomic variants

**Figure 2:**
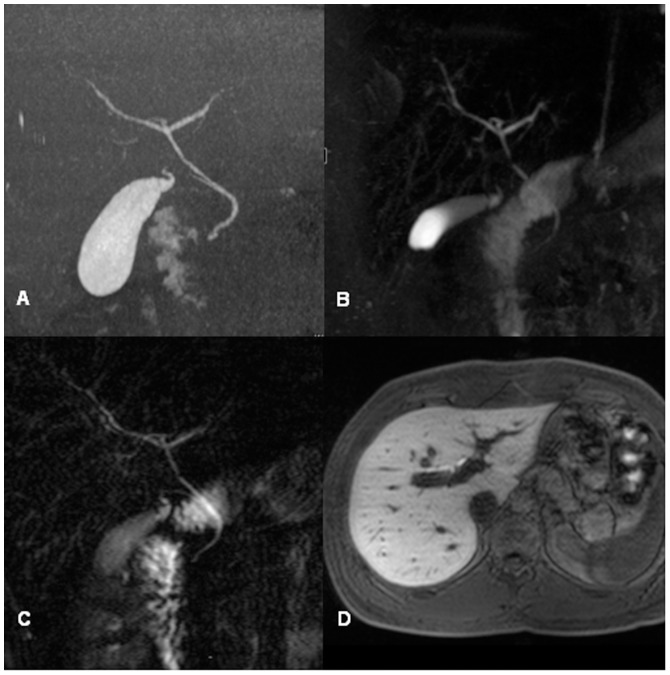
Comparison of MRC sequences in a 23-year-old male potential living liver donor with a central trifurcation (Smadja & Blumgart Type B) seen in contrast enhanced CT cholangiography (A). 3D TSE RESTORE MIP (B), thick slab 2D HASTE (C) and Gd-EOB-DTPA enhanced 3D T1w MRC without IR (D) are all able to detect this variant anatomy.

Anatomic variants were found in 3/30 (10%) patients (Table 4). Two patients presented with a low risk trifurcation (Smadja/Blumgart C2 variant) hiding a drainage of right posterior duct into the ductus hepatocholedochus (DHC) and another one patient had a high risk trifurcation (Smadja/Blumgart D1 variant) with the drainage of the right posterior duct into the left main hepatic duct. Variants were reliably detected with all coronal acquired sequences as well as with axial ce-T1w MRC after coronal reformation (Figure 2). Axial 2D HASTE and BLADE were not able to visualize variants.

**Table 4:** Preoperative assessment of the intrahilar biliary anatomy by MRC versus ce-CTC in n = 30 PLLDs for the right graft ALDLT.

	magnetic resonance cholangiography		ce-CT cholangiography		intraoperative finding	
Patient	Type	Variant	Type	Variant	Type N/	Variant
Nr.	N/A	S & B	N/A	S & B	A	S & B
1. S.S.	N	A	N	A	N	A
2. F.N.	N	A	N	A	na	na
3. P.Ma.	N	A	N	A	N	A
4. P.Mx	N	A	N	A	na	na
5. S.A.	N	A	N	A	N	A
6. L.E	A	C2	A	C2	A	C2
7. N.M.	N	A	N	A	N	A
8. R.D.	N	A	N	A	N	A
9. O.G.	N	A	N	A	na	na
10. D.F.	N	A	N	A	na	na
11. B.P.	N	A	N	A	N	A
12. G.K.	N	A	N	A	na	na
13. B.A.	N	A	N	A	N	A
14. W.G.	N	A	N	A	na	na
15.R.U.	N	A	N	A	N	A
16. R.Ch.	N	A	N	A	na	na
17. O.B-I.	N	A	N	A	N	A
18. F–S.I.	N	A	N	A	N	A
19.Mc. S.	N	A	N	A	N	A
20. B.G.	N	A	N	A	na	na
21.H.U.	A	C2	A	C2	na	na
22. S.F.	N	A	N	A	na	na
23. S.C.	N	A	N	A	na	na
24. S.F.	N	A	N	A	na	na
25. W.B.	N	A	N	A	na	na
26. J.H.	A	D1	A	D1	na	na
27. K.U.	N	A	N	A	na	na
28. H.R.	N	A	N	A	na	na
29. G.S.	N	A	N	A	na	na
30. F.V.	N	A	N	A	na	na

ALDLT: adult-to-adult living liver donor transplantation; PLLD: potential living liver donor; MRC: magnetic resonance cholangiography; ce-CTC: contrast enhanced CT cholangiography; N: normal intrahilar biliary anatomy (bifurcation); A: abnormal intrahilar biliary anatomy (tri-,quadrifurcation); S&B: intrahilar bile duct classification according to Smadja et Blumgart (modified Couinaud).

### Intraoperative results

**Figure 3:**
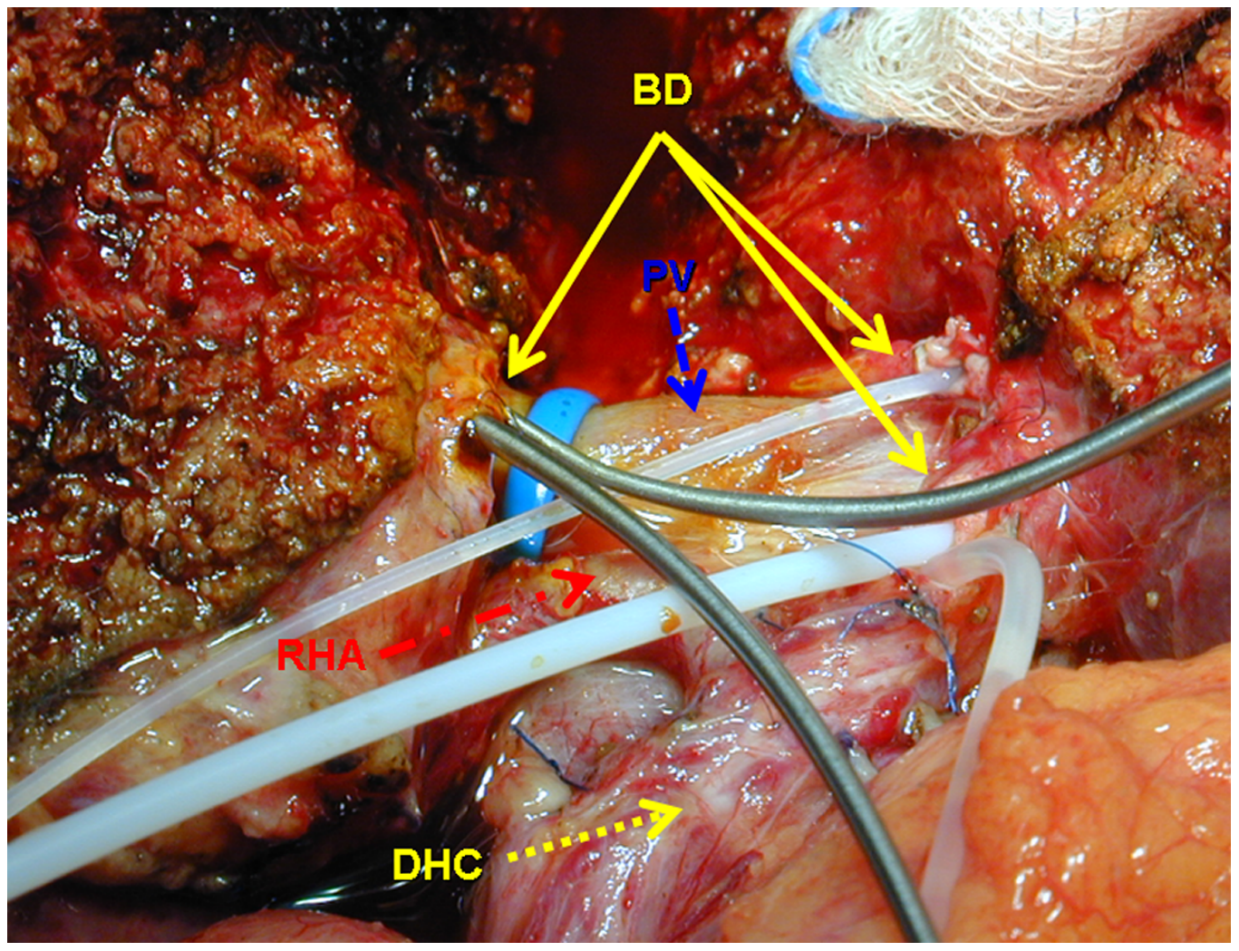
Introperative view of the trans-hilar passage in right graft donor with bile duct trifurcation. A double central bile duct stump (plastic probes) and two bile duct orifices (metal probes) seen along the hilar plate after bile duct transection inside the right hilar corridor. BD: bile duct; DHC: ductus hepatocholedochus; RHA: right hepatic artery; PV: portal vein.

Twelve (40%) PLLDs eventually underwent surgery for right graft donation. There was a 100% correlation between preoperative anatomy estimation on MRI and ce-CTC and intraoperative findings (Figure 3).

### Interobserver agreement

Interobserver agreement for both reviewers proved to be moderate for the axial HASTE sequence concerning quantitative interpretation (0.586). Otherwise, interobserver agreement proved to be good up to excellent (k = 0.622–0.804).

## Discussion

Biliary complications represent the main postoperative morbidity in donors and recipients after right graft ALDLT and in the worst case require a re-transplantation or can lead to a lethal sepsis [20]. The transhilar passage is one of the most difficult surgical steps during the right graft hepatectomy due to extreme variable biliary anatomy [21]. During the transhilar passage the surgeon transects the right bile duct system along the hilar plate (HP) inside a narrow virtual corridor as a part of the right-sided hilar window. The lateral corridor boundary is defined by the right peripheral: sectorial-(2°) or segmental-(3°) branches and the medial boundary is determined by the central-(1°) branching level (see definitions). Hilar transection if performed too much medially hides the risk for inadvertant injury of the left bile duct in the donor whereas complex multiductal reconstructions with more than 2 bile ducts in the graft go along with the increased risk for biliary complications in the recipient [22]. Therefore, for the appropriate donor selection during the preoperative evaluation of the PLLDs and the precise operation planning a 2-steped visualization of the biliary anatomy has been established [23]. The preoperative imaging (first step) is aimed to identify particularly risky anomalies in the donor liver ie. high risk trifurcations (Smadja/Blumgart D1/D2 variants) with the cross over drainage of the right posterior/anterior duct into the left main hepatic duct likewise complex quadrifurcations (Smadja/Blumgart E1/E2 variants) in order to protect the donor from injuries and also to prevent dangerous biliary reconstructions in the graft which can result in postoperative bile leakage or anastomotic stricture [24]. During the donor operation (second step) an intraoperative cholangiography has been mostly performed prior to the transhilar passage in order to determine the transection line inside the corridor marked with a clip along the hilar plate inbetween the central-1° and right peripheral 2°/3° branching levels [13]. Therefore, a sufficient imaging allowing for the proper donor selection and reliably assisting the transhilar passage provides the 3-dimensional visualisation of the bile duct anatomy inside and outside the corridors but in topographical relation to the individual segmental liver make up [12].

The purpose of this study was to compare different MR cholangiography sequences in potential living liver donors (PLLDs) to contrast enhanced CT cholangiography. Various recent evaluations as well as our own experience show that ce-CTC can be regarded as a complementary imaging modality to direct (e.g. intraoperative) cholangiography techniques and therefore provided a non-invasive diagnostic reference standard for the evaluation of the biliary anatomy of PLLDs in our institution [11], [25]. Moreover, the considerable drawback of intraoperative cholangiography: its “one-dimensionality” was in our experience satisfactory complemented by the 3-dimensional CT cholangiography exactly related to the individual segmental liver map provided by the software assistant HepaVision (MeVis, Bremen, Germany).

This study carries two messages we believe to be of value in the preoperative evaluation of PLLDs and operation planning:

First, all analyzed MRC sequences are still inferior compared to ce-CTC. This is been underlined by Hyodo et al. [26] who compared MRC and CTC in the perioperative setup: They concluded that CTC as well as MRC are minimally invasive methods that provide precise depiction of the biliary system. While CTC is already and will be in the future even more limited to certain countries due to the availability of the CT contrast agents, MRC has proven its applicability for the evaluation of extrahepatic as well as dilated intrahepatic bile ducts, but has to improve for the intrahepatic, non-dilated bile ducts which can usually be found in healthy PLLDs.

Second, the most useful MR cholangiography sequences were 3D T2 TSE and SPACE sequences including the calculated maximum intensity projections which have been already reported as best MRC sequences in healthy volunteers [27]. Morita et al. compared these 3D sequences and found that the SPACE sequence was superior to the conventional sequence, which we cannot prove in our trial in PLLDs. The next best MRC sequences were 2D MRC sequences including thick-slab HASTE sequences. Here, our results are in accordance with different other groups comparing MRC sequences: Choi et al. [28] compared 2D and 3D sequences in patients with malignant biliary obstruction. They also found higher image quality for the 3D sequences without a difference concerning accuracy. The group of Basaran et al. compared source and MIP images of a T2 TSE sequences and found no differences concerning specificity, sensitivity and positive as well as negative predictive values [13]. Lim et al. analyzed MRCP for preoperative evaluation of PLLDs using 2D T2w MRC, 3D T2w MRC, and 3D contrast-enhanced T1w MRC [29]. They found that 3D T2w MRC provided superior biliary visualization than 2D T2w MRC and 3D contrast-enhanced T1w MRC. Their results are in concordance with ours: They found to be the 3Dw sequences most valuable, followed by 2D sequences and T1w contrast-enhanced MRCP. The less good performance of contrast enhanced T1 weighted MRCP sequences is also reflected in other studies: Frydrychowicz et al. [30] concluded in their comparative study in patients with primary sclerosing cholangitis that T1 weighted MRCP using Gd-EOB-DTPA can be useful adjunct but cannot replace T2 weighted MRCP sequences. They found that T1w MRCP is inferior to T2w MRCP in the evaluation of the intrahepatic bile ducts, but can provide useful additional information for central and extrahepatic biliary anatomy. An et al. [31] found that gadobenate dimeglumine enhanced MRCP was more accurate than T2 weighted MRCP in the preoperative bile duct evaluation of living liver donors. They (apart from us) only used rapid thick and thin slab 2D turbo spin echo sequences for T2w MRCP. Furthermore, they used gadobenate dimeglumine. Nevertheless, results of the study group of Lee et al. suggest that there are no significant differences regarding gadobenate dimeglumine and Gd-EOB-DTPA for gadolinium-enhanced T1w MRCP as they showed in their study in 2011 [18]. In contrast to these results, Mangold et al. found a significantly higher image quality for Gd-EOB-DTPA enhanced T1w MRCP compared to conventional T2w images. They did not specify in their work if a 2D or 3D T2w sequence was used for comparison.

Due to recent developments in sequence and magnetic field strengths, one can argue why our study was performed at 1.5T and not at higher field strengths. Kim et al. were able to show that 3T MRCP compared to 1.5T MRCP did not significantly increase accuracy for identification of biliary anatomy. Therefore, the clinical gold standard is still 1.5 T MRCP.

In conclusion we could show that different MRCP sequences are helpful to evaluate biliary anatomy in the preoperative work-up of PLLDs. Source and MIP images of 3D T2w sequences provide the best knowledge of the bile ducts. These sequences can be supplemented by 2D T2w MRCP sequences. Gd-EOB-DTPA enhanced T1w MRCP sequences can be of additional value to characterize central and extrahepatic bile ducts. As Gd-EOB-DTPA can also be used as dynamic contrast agent for vascular analysis, the agent can be used as “all-in-one”-contrast agent in the preoperative evaluation of PLLDs.
